# Addendum to “A Hyperbolic View of the Seven Circles Theorem”

**DOI:** 10.1007/s00283-022-10231-9

**Published:** 2023-01-28

**Authors:** Kostiantyn Drach, Richard Evan Schwartz

**Affiliations:** 1 Institute of Science and Technology Austria, Am Campus 1, 3400 Klosterneuburg, Austria; 2 Brown University, 151 Thayer Street, Providence, RI 02912 USA

The authors would like to draw the reader’s attention to a YouTube video^1^ by Benedikt Hahn, Paulina Hering, and Pavel Zwerschke that provides a crash course in hyperbolic geometry and presents a beautifully animated proof of the seven circles theorem, based on our article “A Hyperbolic View of the Seven Circles Theorem,” which appeared recently in this journal.^2^

## Footnotes

1 Available at https://youtu.be/m9v0h2ibYpo.

2 *Math. Intelligencer* 42:2 (2020), 61–65. https://doi.org/10.1007/s00283-019-09952-1.

